# No Evidence for a Boost in Psychosocial Functioning in Older Age After a 6-Months Physical Exercise Intervention

**DOI:** 10.3389/fnhum.2022.825454

**Published:** 2022-03-11

**Authors:** Sandra Düzel, Johanna Drewelies, Sarah E. Polk, Carola Misgeld, Johanna Porst, Bernd Wolfarth, Simone Kühn, Andreas M. Brandmaier, Elisabeth Wenger

**Affiliations:** ^1^Center for Lifespan Psychology, Max Planck Institute for Human Development, Berlin, Germany; ^2^Department of Psychology, Humboldt University Berlin, Berlin, Germany; ^3^International Max Planck Research School on the Life Course (LIFE), Berlin, Germany; ^4^Department of Sports Medicine, Charité–Universitätsmedizin Berlin and Humboldt Universität zu Berlin, Berlin, Germany; ^5^Lise Meitner Group for Environmental Neuroscience, Max Planck Institute for Human Development, Berlin, Germany; ^6^Neuronal Plasticity Working Group, Department of Psychiatry and Psychotherapy, University Medical Center Hamburg-Eppendorf, Hamburg, Germany; ^7^Max Planck UCL Centre for Computational Psychiatry and Ageing Research, Berlin, Germany; ^8^Department of Psychology, MSB Medical School Berlin, Berlin, Germany

**Keywords:** aging, psychosocial functioning, aerobic exercise, bicycle ergometer training, perceived stress, future time perspective, well-being, loneliness

## Abstract

The beneficial effects of physical exercise on physical health and cognitive functioning have been repeatedly shown. However, evidence of its effect on psychosocial functioning in healthy adults is still scarce or inconclusive. One limitation of many studies examining this link is their reliance on correlational approaches or specific subpopulations, such as clinical populations. The present study investigated the effects of a physical exercise intervention on key factors of psychosocial functioning, specifically well-being, stress, loneliness, and future time perspective. We used data from healthy, previously sedentary older adults (*N* = 132) who participated in a 6-month at-home intervention, either engaging in aerobic exercise or as part of a control group who participated in foreign language-learning or reading of selected native-language literature. Before and after the intervention, comprehensive cardiovascular pulmonary testing and a psychosocial questionnaire were administered. The exercise group showed significantly increased fitness compared to the control group. Contrary to expectations, however, we did not find evidence for a beneficial effect of this fitness improvement on any of the four domains of psychosocial functioning we assessed. This may be due to pronounced stability of such psychological traits in older age, especially in older adults who show high levels of well-being initially. Alternatively, it may be that the well-documented beneficial effects of physical exercise on brain structure and function, as well as cognition differ markedly from beneficial effects on psychosocial functioning. While aerobic exercise may be the driving factor for the former, positive effects on the latter may only be invoked by other aspects of exercise, for example, experiences of mastery or a feeling of community.

## Introduction

There is convincing evidence from numerous studies that physical exercise may be beneficial for physical and cognitive functioning (e.g., Prakash et al., 2015; Macpherson et al., 2017). First and foremost, physical activity has been shown to lower the risk for cardiovascular disease, stroke, and diabetes, as well as reduce all-cause mortality overall, thereby leading to higher life expectancy (Lear et al., 2017). Observational studies also continue to suggest that adults who engage in physical activity have a reduced risk of cognitive decline and dementia (Podewils et al., 2005; Ferencz et al., 2014), and that exercise can exert a protective effect on cognitive functioning (Ahlskog et al., 2011; Ngandu et al., 2015). Importantly, these documented beneficial effects of physical activity span from pure physiological effects to higher-order cognition as described above, as well as to anatomy (Firth et al., 2018) and functional connectivity of the brain (Stillman et al., 2019). The common conception of the potential positive effects of physical exercise on brain structure and function relies on the following assumed mechanism of action: physical exercise targets the cardiovascular system and may therefore lead to increased brain perfusion, resulting in better oxygenation of all cells in the brain (Pereira et al., 2007). This in turn may lead to maintained or even improved indices of brain structure, as angiogenesis may trigger neurogenesis, synaptogenesis, or changes in neuropil, all leading to increased gray matter volume (Kleemeyer et al., 2015; Maass et al., 2015).

Even though it has been shown that exercise is related to several physiological outcomes, evidence of its effect on mental health, mood, or psychosocial functioning in general in healthy older adults is still scarce or inconclusive. Psychosocial functioning is used here as an umbrella term for a number of psychological risk and resilience factors that are related but distinct. Importantly, as such it cannot be measured as a single construct or factor but rather encompasses distinct, established and validated measures, such as well-being or perceived stress. The lack of experimental evidence for a causal link between physical activity and improved psychological functioning has been noted in various qualitative reviews of studies on the aging population [see Brown (1992), O’Connor et al. (1993), McAuley and Rudolph (1995), and Spirduso and Cronin (2001)]. Research on the association of physical activity and psychosocial functioning generally report positive effects (Netz et al., 2005; Penedo and Dahn, 2005). For example, one meta-analysis reported a small but significant effect of exercise on well-being in healthy older adults (Cohen’s *d* = 0.24; Netz et al., 2005). However, many current findings are based on correlational designs that have limited value in informing about causal effects and, particularly, effects at the within-person level. Additionally, studies are often based on a variety of exercise regimes, such as regimes including dancing, Hatha yoga, or group-based activities (West et al., 2004), thereby confounding which factors could specifically be driving the positive effects of engaging in exercise. Other studies did not investigate psychosocial functioning as a multi-faceted construct, but rather only focused on select aspects, often also in clinical populations (e.g., McAuley and Rudolph, 1995; Langlois et al., 2013). Therefore, here we focus on four global facets of psychosocial functioning which have previously been shown to be associated with physical exercise or fitness. Specifically, we examine exercise- and fitness-related effects on well-being, perceived stress, loneliness, and future time perspective. Different factors may play a role in the suggested positive effects of physical exercise on psychosocial functioning. These factors may exist on the level of physiology (e.g., *via* changes of catecholamines, endorphins), on the level of psychology (e.g., distraction, feeling of mastery), and on the level of the environment (e.g., social integration).

Well-being has consistently been shown to be associated with higher levels of physical exercise (Hassmén et al., 2000; McAuley et al., 2007; Zubala et al., 2017; Mandolesi et al., 2018). However, several open questions remain regarding this association, including the directionality of effects, as well as whether the effects occur at specific doses of exercise. The time scale at which these effects emerge is also not yet fully understood, as well as whether or not the effects of exercise are preventive or corrective (Scully et al., 1998).

Perceived stress, that is, the subjective perception of stress (Salmon, 2001; Klaperski et al., 2013; Lippke et al., 2015), can also be influenced by physical activity. This is notable as the biological and behavioral consequences of experiencing high levels of stress have been shown consistently to have a serious impact on several physical health issues (Cohen et al., 2007; Haskell et al., 2007; Miller et al., 2009). Moreover, it has been demonstrated that the physical health benefits of physical activity are particularly evident among individuals who experience high, as compared to low levels of stress (Brown and Siegel, 1988; Carmack et al., 1999). Physical training interventions have been repeatedly shown to reduce the burden of chronic stress (McEwen, 2007) and can also lead to significant changes in acute stress levels (Atlantis et al., 2004).

Loneliness can be described as the subjective experience that one’s social network is insufficient in size or unsatisfactory in quality (de Jong-Gierveld, 1987). Loneliness is associated with a number of negative physical health outcomes, as well as impaired cognition, clinical dementia, and depression (Ehlers et al., 2017). In a large sample of older adults, Netz et al. (2013) found that perceptions of loneliness were highest among sedentary participants, while participants categorized as “sufficiently active” (involved in moderate physical activity for at least 150 min a week or in intensive activity for at least 75 min a week) reported lower levels of loneliness. Similarly, physical intervention studies have been shown to reduce loneliness in older adults (McAuley et al., 2000; Masi et al., 2011). Social support environment and physical exercise may jointly serve to reduce perceptions of loneliness (Masi et al., 2011; McEwen and Gianaros, 2011; McHugh and Lawlor, 2012).

Future time perspective (FTP) relates to the human cognitive capacity for imagining the future, and forms an important psychological basis of motivated behavior in everyday life (Andre et al., 2018). Time perspective, a psychological construct denoting subjective orientation toward the remaining future horizon, has been consistently associated with health behaviors; higher FTP scores have been associated with more frequent exercise (Adams and Nettle, 2009; Gulley, 2013; Guthrie et al., 2013; Hall and Epp, 2013). However, most studies examining the association between exercise and time perspective were based on correlational designs, and it remains unclear whether and how exercise directionally affects FTP.

### The Present Study

Using data from an intensive training study with 132 healthy, previously sedentary older adults that included a physical exercise regime, foreign language learning, and a book club, we investigated the effects of 6 months of physical exercise training on four major aspects of psychosocial function. Specifically, we expected that engaging in physical activity would lead to an increase in well-being, and would ameliorate perceived acute stress and perceived loneliness. Finally, we expected that physical exercise would extend one’s future time perspective.

## Materials and Methods

### Participants

Volunteers were recruited *via* newspaper advertisements and previous participation in studies [Berlin Aging study II; see Gerstorf et al. (2015)]. Two hundred and one participants met the following inclusion criteria: right-handed, age between 63 and 78 years old, no history of head injury, serious medical, neurological, or psychological disorder, or use of any medication affecting memory function, suitable for an MR environment (e.g., no metallic implants, claustrophobia), could not fluently speak a romance language and were not proficient in more than one language besides German, and did not engage in aerobic exercise more than once every 2 weeks prior to study enrollment. Those were sent to a medical check-up. Twenty-two participants were excluded due to contraindication for interval-based at-home (i.e., unsupervised) exercise training (e.g., aneurysm, insufficiently treated high blood pressure, etc.). One hundred and fifty-nine participants were enrolled in the study. During training, 17 participants dropped out due to health-related issues or time constraints, leaving 142 participants, who completed the full study. Participants were considered fully compliant if they completed at least a total of 1890 min (equaling approximately 90 min per intervention week) of study-related activity during the 6-month study period. Following this screening, the final effective sample consists of 132 participants (63–78 years old; *M* = 70.6, SD = 3.7; female = 50.8%) in the current analyses. The Ethics committee of the German Society for Psychology (DGPs) approved the study and written informed consent was collected from all participants.

### Experimental Design

Our 6-month intervention study originally aimed to investigate differential effects of foreign language training, exercise training, and a combination of the two on brain structure and function and cognitive performance in older adults. A more detailed description of the methods and all collected variables can be found in Wenger et al. (2022).

All eligible volunteers were pseudo-randomly assigned to one of four groups: (1) an active control group, (2) a language training group, (3) an exercise training group, or (4) a combined language and exercise training group. Before the 6-month training period (pre-test), participants underwent a comprehensive physical assessment including cardiopulmonary exercise testing (CPET) with lactate diagnostics, cognitive testing, a magnetic resonance imaging (MRI) session, and were asked to fill out comprehensive psychosocial take-home questionnaires and return these during their next visit to the institute, before beginning the intervention (pre-test). After 3 months (mid), participants underwent cognitive testing and MR imaging again (mid). After the full training period of 6 months (post-test), CPET, cognitive testing, MR imaging, and psychosocial take-home questionnaires were administered again.

### Interventions

#### Active Control Group

The active control group (ACG; *n* = 32) was instructed to read pre-selected German literature on a tablet (Lenovo TB2-X30L TAB) for about 45 min per day for at least 6 days per week. Additionally, participants attended weekly group sessions during which they discussed short stories and poems informally and without preparation, selected and verbally presented by external facilitators^1^.

#### Language Group

The language group (LG; *n* = 32) was instructed to learn Spanish as a novel foreign language within the 6-month intervention period using the Babbel application on a tablet, studying for about 30 min a day (or until they completed one lesson) for 6 days a week at home. Participants were also instructed to read pre-selected German literature on the tablet at a slow pace for an additional 15 min a day, in order to spend a total of ca. 45 min doing study-related activities each day. They also participated in weekly 1-h group sessions (5–10 participants per session) consisting of a Spanish class led by external Spanish teachers.

#### Exercise Group

Participants in the exercise group (EG; *n* = 39) engaged in moderate aerobic exercise at home three to four times per week on a bicycle ergometer (DKN Ergometer AM-50) and Bluetooth-linked tablet with a personalized interval training regime. The training initially lasted 30 min at an individually set intensity, and the difficulty increased by 3 min per interval and by 3 to 4 watts approximately every 2 weeks. After completing a training session, participants could indicate if they found the training too easy or difficult, and the intensity could be remotely adjusted accordingly in order to keep training intensity at a reasonable difficulty. Participants in the exercise group were also instructed to read pre-selected German literature on the tablet for an additional 15 min on days when they trained and for 45 min on the other days, again in order to spend a total of ca. 45 min doing study-related activities each day. Finally, those in the exercise group participated in weekly 1-h group sessions consisting of toning and stretching, led by an external instructor.

#### Combined Language and Exercise Group

Participants in the combined language and exercise training group (L + EG; *n* = 29) learned Spanish using Babbel 6 days per week and also engaged in the interval training on an ergometer for 3–4 days per week, as described above. They also joined weekly Spanish classes (separate from the language group) in groups.

#### Analysis Based on a Division of All Exercisers vs. All Non-exercisers

In this analysis, we specifically wanted to investigate any potential effects of physical exercise on measures of psychosocial functioning. As described above, we ensured that participants in all groups spent comparable amounts of time on study-related activities, that is, both the exercise-only group and the combined language and exercise group were instructed to spend equal amounts of time exercising on their bicycle ergometers. Therefore, it was possible to collapse all participants who trained on their bicycle ergometers into an exercisers group (EG & L + EG; *n* = 68) and compare these to a non-exercisers group, which included all participants who did not do any kind of physical exercise training (ACG and LG; *n* = 64).

### Fitness Assessment

The physical assessment with CPET was used to obtain participants’ initial fitness level as a basis of the individual training plans. The physical assessment lasted about 2 h and consisted of a general check-up of physical capability for participation in the study at baseline, including a comprehensive anamnesis, a physical check-up, measurement of height, weight, body composition, and lung function, and bicycle ergometry with electrocardiogram and lactate diagnostics. To determine aerobic fitness, peak oxygen uptake (VO_2_peak) was assessed using CPET by performing a graded maximal exercise test on a cycling ergometer. This was done at pre-test, and again after 6 months at post-test. After the pre-test measurements, an individual training plan was given to each participant, which was the basis for participants’ interval training at home.

### Measures of Psychosocial Functioning

A set of self-report questionnaires of psychosocial functioning that were previously translated into German, validated, and applied within the BASE-II study sample [see Gerstorf et al. (2015)] were administered in the current sample. This psychosocial assessment comprised the following measures of psychosocial functioning.

#### Well-Being

We used the Satisfaction with Life Scale to assess the cognitive-evaluative component of well-being (Cronbach’s α = 0.88; Diener et al., 1985), in which participants rated five statements (e.g., *“In most ways my life is close to my ideal”*) on a five-point scale ranging from 1 (*strongly disagree*) to 5 (*strongly agree*) to indicate their level of agreement. Two participants did not provide data for well-being at pre-test, and five did not provide data for well-being at post-test.

#### Perceived Stress

We used the eight-item version of the Perceived Stress Scale (PSS) to assess perceived stress within the last four weeks (Cronbach’s α = 0.85; Cohen et al., 1994). Participants rated eight statements (e.g., in the last month, “*how often have you felt nervous and stressed?*”) on a five-point scale ranging from 0 (*never*) to 4 (*very often*) to indicate their level of agreement. Six participants did not provide data for perceived stress at pre-test, four did not provide data at post-test.

#### Loneliness

Loneliness was measured with the UCLA Loneliness Scale (Russell, 1996) consisting of seven items (Cronbach’s α = 0.81; Hülür et al., 2016). Participants were asked to rate each statement on a five-point Likert scale ranging from 1 (*strongly disagree*) to 5 (*strongly agree*). Higher scores indicate stronger feelings of loneliness. Three participants did not provide data for loneliness at pre-test, four did not provide data at post-test.

#### Future Time Perspective

We used the 10-item Future Time Perspective scale (Carstensen and Lang, 1996) to quantify future time perspective (Cronbach’s α = 0.88; Kozik et al., 2020). Participants responded to each item (e.g., “*Many opportunities await me in the future.*”) using a Likert scale ranging from 1 (*very untrue for me*) to 7 (*very true for me*). The scale composite consists of the unit-weighted mean across items, with lower scores indicating a more limited general future time horizon. Four participants did not provide data for FTP at pre-test, five did not provide data at post-test.

For each measure, the mean over all items was calculated and used in the following analyses.

### Covariates

#### Socio-Demographic Variables

Participants’ age was calculated as years from birth to the first cognitive assessment. Sex was indicated by a dichotomous variable (male; female). Education was indicated by the number of years in formal schooling (range 9–18 years).

### Statistical Analysis

Statistical analyses were conducted in JASP (JASP Team, 2021, version 0.15^2^). All dependent variables, that is, VO2_peak_, well-being, perceived stress, loneliness, and FTP met the criteria for normal distribution. Correlations were calculated between all variables. We applied a significance threshold of *p* < 0.05 to all frequentist analyses and report the effect size omega squared (ω^2^), where ω^2^ = 0.01 indicates a small effect, 0.06 indicates a medium effect, and 0.14 indicates a large effect.

First, to examine the effectiveness of the exercise intervention, we assessed improvements in physical fitness during the intervention period. We performed a classical two-way repeated-measures analysis of variances (ANOVA) with group (all exercisers vs. all non-exercisers) as between-subject factor and time (pre-test, post-test) as a within-person factor, and VO_2_peak as the outcome variable, adjusting for age, sex, and education. Follow-up paired *t*-tests were performed to assess within-group changes in fitness levels over time. We additionally performed a Bayesian repeated measures ANOVA with the factors group and time.

Secondly, to answer our main research question, we examined the effect of exercise-training on psychosocial functioning. We performed four classical two-way repeated-measures ANOVAs with group (all exercisers vs. all non-exercisers) and time (pre, post) as factors and well-being, perceived stress, loneliness, and future time perspective as outcome variables, adjusting for age, sex, and education, as well as Bayesian repeated measures ANOVAs with the factors time and group and report our results based on the nomenclature of Kass and Raftery (1995). Differing degrees of freedom (*df*s) reflect missing data for some variables as described above).

Lastly, we investigated whether interindividual differences in fitness change were associated with changes in any of the psychosocial functioning measures, irrespective of group assignment. To this end, we performed correlations between change in VO_2_peak and change in well-being, perceived stress, loneliness, and time perspective, respectively, across all participants, accounting for age, sex and education.

To put our findings into perspective, we carried out a power analysis for a within-between interaction of a repeated-measures ANOVA with two time points and two groups with a total sample size of 132, an α-error probability of 0.05 and a power (1-β error probability) of 0.95. To do so, we used the G*Power toolbox^3^ (Faul et al., 2007) with the effect size specification *f*^2^ as in Cohen (1988), where 0.02 represents a small effect, 0.15 represents a medium effect, and 0.35 represents a large effect (Cohen, 1988).

## Results

### Sample Characteristics

Descriptive statistics of the sample demographics and intervention specifics per group are provided in Table 1. There were no significant differences between all non-exercisers and all exercisers in terms of age, years of education, sex, or total intervention time. Table 2 summarizes the intercorrelations between the selected psychosocial variables and covariates across all groups. Across all groups, sex was significantly negatively associated with years of education with women having fewer years of education (*r* = −0.307, *p* < 0.001) and fitness (VO_2_peak) at baseline (*r* = −0.406, *p* < 0.001) and at post-test (*r* = −0.445, *p* < 0.001), with men showing higher VO_2_peak values. At pre-test, well-being was, as expected, negatively associated with perceived stress (*r* = −0.396, *p* < 0.001) and loneliness (*r* = −0.265, *p* < 0.05), and positively associated with FTP (*r* = 0.525, *p* < 0.001). FTP was negatively associated with perceived stress (*r* = −0.213, *p* < 0.05) and loneliness (*r* = −0.219, *p* < 0.05). There were no significant associations between fitness and well-being, perceived stress, loneliness or FTP at baseline. These were also no significant group differences in fitness, well-being, perceived stress, loneliness, or FTP at baseline (all *p*s > 0.05). All repeated measures showed very high within-measurement correlations over time (VO_2_peak: *r* = 0.839, well-being: *r* = 0.733, perceived stress: *r* = 0.684, loneliness: *r* = 0.702, FTP: *r* = 0.760; all *p*s < 0.001).

**TABLE 1:** Sample descriptives and intervention specifics.

	All non-exercisers		All exercisers		Difference between two groups	
	Reading group	Language group	Exercise group	Exercise + language group		
*n* fully compliant	32	32	39	29		
Age, M ± SD (range)	70.75 ± 3.93 (63.98–76.03)	71.54 ± 3.67 (65.74–78.07)	69.78 ± 3.47 (63.87–76.90)	70.63 ± 3.45 (64.35–77.23)	*F*(1,130) = 2.54	*p* = 0.11
Sex,% females	40.63%	56.25%	51.28%	51.72	χ^2^(1) = 0.12	*p* = 0.73
Years of education, M ± SD (range)	13.44 ± 3.15 (7–16)	13.15 ± 3.31 (7–16)	13.45 ± 3.03 (7–16)	13.59 ± 3.48 (7–16)	*F*(1,126) = 0.14	*p* = 0.71
Total min of intervention, M ± SD (range)	4771.75 ± 1819.59 (2505.00–10858.00)	6285.01 ± 2566.73 (2250.23–11718.70)	6269.10 ± 1555.17 (2406.00–9790.00)	6275.46 ± 2863.84 (1895.12–12851.85)	*F*(1,130) = 3.56	*p* = 0.06
Minutes spent reading, M ± SD (range)	4771.75 ± 1819.59 (2505.00–10858.00)	1756.06 ± 1371.17 (30.00–5227.00)	3258.80 ± 1212.46 (915.00–5855.00)	—		
Minutes spent language learning, M ± SD (range)	—	4528.94 ± 1834.32 (1870.60–8643.58)	—	3995.84 ± 2334.65 (823.12–8697.85)		
Minutes spent exercising, M ± SD (range)	—	—	2966.47 ± 664.51 (1042.00–4101.00)	2418.71 ± 945.66 (99.98–4296.01)		

**TABLE 2:** Intercorrelations of study measures before and after the intervention across all groups.

Variable	1.	2.	3.	4.	5.	6.	7.	8.	9.	10.	11.	12.	13.	14.
1. Age	—													
2. Education	0.056	—												
3. Sex	−0.074	−0.307*	—											
4. Time	0.164	0.033	0.052	—										
5. VO_2__pre	−0.170	0.163	−0.406*	0.149	—									
6. WB_pre	−0.007	0.075	−0.140	−0.071	0.143	—								
7. Stress_pre	0.014	0.079	0.138	0.082	−0.028	−0.396*	—							
8. Lone_pre	−0.026	0.031	−0.029	−0.119	−0.025	−0.265*	0.103	—						
9. FTP_pre	−0.111	−0.092	−0.040	−0.060	0.144	0.525*	−0.213*	−0.219*	—					
10. VO_2__post	−0.178*	0.189*	−0.445*	0.147	0.839*	0.171	−0.141	−0.039	0.147	—				
11. WB_post	−0.130	−0.025	−0.038	−0.065	0.140	0.733*	−0.303*	−0.330*	0.563*	0.146	—			
12. Stress_post	0.029	0.153	0.014	0.031	−0.006	−0.463*	0.684*	0.074	−0.186*	−0.117	−0.462*	—		
13. Lone_post	0.033	0.052	0.013	−0.024	−0.024	−0.316*	0.163	0.702*	−0.297*	−0.087	−0.449*	0.205*	—	
14. FTP_post	−0.085	−0.206*	0.057	−0.147	0.042	0.483*	−0.271*	−0.204*	0.760*	0.056	0.528*	−0.265*	−0.302*	—

sex: 1 = male, 2 = female; VO_2_, peak oxygen uptake; time, total time spent on study-related activities (reading, exercising, language learning); WB, well-being; stress, perceived stress; lone, loneliness; FTP, future time perspective. *Correlation is significant at the 0.05 level (2-tailed).

### Effect of an Exercise Intervention on Psychosocial Functioning

**FIGURE 1:**
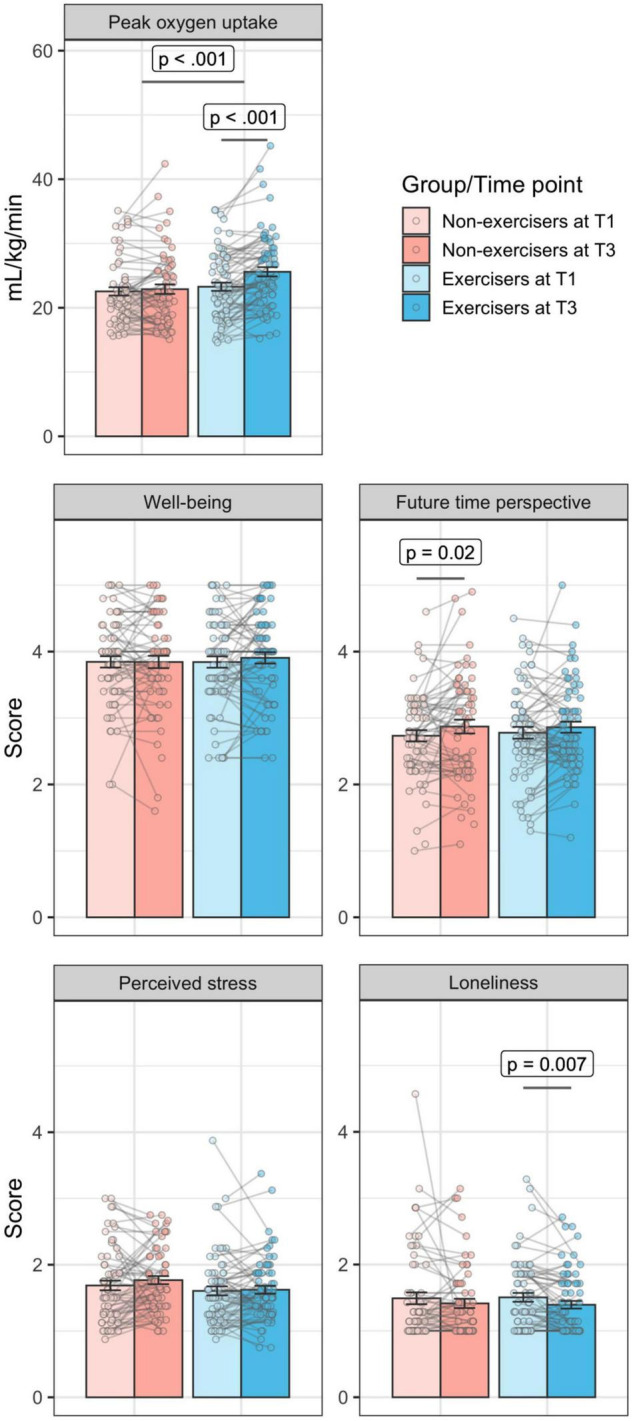
A significant change over time in peak oxygen update (VO_2_peak) in all exercisers (displayed in blue) in comparison to all non-exercisers (displayed in red). Contrary to expectations, there was no significant time by group interactions in well-being, perceived stress, loneliness, or future time perspective. Error bars represent ± 1 standard error (SE).

A two-way repeated-measures ANOVA showed a significant group-by-time interaction in fitness (VO_2_peak), *F*(1,118) = 11.29, *p* < 0.001, ω^2^ = 0.009 (see Figure 1). Similarly, a Bayesian repeated measures ANOVA indicated decisive evidence for the inclusion of the main effect of time (BF_Inclusion_ = 25514.883) and strong evidence for the inclusion of a main effect of group (BF_Inclusion_ = 21.043), as well as strong evidence (Kass and Raftery, 1995) for the inclusion of the interaction effect between group and time (BF_Inclusion_ = 59.200). Together, this shows that our at-home bicycle ergometer training intervention did improve participants’ fitness in an objective fitness measure, in comparison to a control group.

Contrary to our expectations, repeated measures ANOVAs revealed no significant differences between the two groups in change over time in any of the indicators of psychosocial functioning (well-being, perceived stress, loneliness, and FTP), adjusting for age, sex, education: well-being, *F*(1, 119) = 0.53, *p* = 0.47, ω^2^ = 0.00; perceived stress, *F*(1, 115) = 0.58, *p* = 0.45, ω^2^ = 0.00; loneliness, *F*(1, 118) = 1.98, *p* = 0.16, ω^2^ = 0.00; FTP, *F*(1, 116) = 0.33, *p* = 0.57, ω^2^ = 0.00 (see Figure 1). Bayesian repeated measures ANOVAs corroborate our conclusions about the group-by-time interaction in all of the measures, with evidence against the inclusion of an interaction term in the ANOVA with varying strength: evidence against the inclusion of the interaction in wellbeing was strong BF_Inclusion_ = 0.052, in perceived stress was strong, BF_Inclusion_ = 0.066, in loneliness was strong, BF_Inclusion_ = 0.088, and in FTP was substantial, BF_Inclusion_ = 0.229. Further details on the results of the Bayesian ANOVAs can be found in the Supplementary Material.

A power analysis showed that with a total sample size of 132, two timepoints and two groups, an α-error probability of 0.05 and power of 0.95, the target effect size that could have been detected would be *f*^2^ = 0.316. This means that we expect to be able to detect a medium to large effect with our repeated measures ANOVAs, testing for an interaction between group and time, in 95 out of 100 times.

In an additional analysis, we investigated whether improvements in fitness were associated with changes in psychosocial functioning across all participants, irrespective of groups. Here, we correlated the difference from pre-test to post-test in VO_2_peak with the differences in well-being, perceived stress, loneliness, and time perspective, accounting for age, sex, and education. No significant associations were found (all *p*s > 0.05).

## Discussion

In this study, we investigated the effects of a fitness intervention on major psychosocial constructs such as well-being, perceived stress, loneliness, and future time perspective. We used data from healthy, previously sedentary older adults who completed a 6-month at-home intervention in either a reading, a learning-only, an exercise-only, or a combination (exercise and language-learning) group. All participants underwent comprehensive physical assessments with CPET and completed a take-home psychosocial questionnaire before and after the intervention. We compared the effects of our exercise intervention with the effects of other non-sports-related activities, namely foreign language learning, and a book club.

Results indicated that fitness significantly increased within those participants that did at-home bicycle ergometer training compared to all non-exercisers. Contrary to our hypotheses, we did not find evidence for an improvement in psychosocial functioning as a result of our longitudinal exercise intervention. In the following, we discuss possible reasons why no significant effects of physical exercise on the established constructs of well-being, perceived stress, loneliness, and future time perspective were observed.

Adult development and aging are characterized by stability in many domains of psychosocial functioning. Even though there is a shift in the gain-loss dynamic in older age (Baltes, 1987), older individuals can compensate for those losses, potentially by adapting their goals or focusing on positive and meaningful aspects of life (e.g., Charles et al., 2001; Charles and Carstensen, 2010; Heckhausen et al., 2010). Importantly, after experiencing both negative or positive life events, older individuals seem to experience short-term fluctuations but return to their emotional equilibrium (Helson, 1964; Hülür and Gerstorf, 2018). This phenomenon of returning back to a person-specific equilibrium has been discussed under the concept of set-point theory for different measures of psychosocial functioning (Luhmann and Intelisano, 2018), and also concerning body weight and exercise (Müller et al., 2018). Even though the claim of set-point theory in the context of subjective well-being that it should in principle apply to each and every individual except for severe exceptional cases has also been challenged (e.g., Headey, 2013; Headey et al., 2021), it may still be that those older adults with specific personality traits tend to stay within their known emotional boundaries. Relatedly, it is also possible that not exercising itself but rather personal meaningful (physical) activities affect psychosocial functioning. Future research needs to examine the role of importance and degree of freedom in choosing activities when examining the associations between physical exercise and psychological functioning.

Additionally, within the scope of the current analysis, it is not possible to interpret at which time scale the effects of exercise on psychosocial functioning may play out in the long term. There are potentially no immediate effects of exercise on psychosocial functioning, but rather, exercise might manifest in better physical health over a longer period of time, for example, by slowing disease progression or general physical decline. These effects on physical health may in turn result in benefits to psychosocial functioning at a later point in time.

Although we aimed to examine many facets of psychosocial functioning, these four selected concepts of well-being, perceived stress, loneliness, and future time perspective still only cover specific aspects of psychosocial functioning. Thus, we cannot say whether the current findings generalize to other aspects of psychosocial functioning, e.g., life goals and goal engagement, or control beliefs. We also did not assess personality traits in this study. However, as previous studies have shown an association between physical activity and personality development over longer periods, ranging from 4 to 10 years (Allen and Laborde, 2014; Stephan et al., 2014; Allen et al., 2017), it would be interesting to investigate whether a 6-month long physical intervention study in previously sedentary older adults could also have an influence on dimensions of personality.

The strict inclusion criteria in our study, mainly due to the MRI-compatibility requirements and the generally high requested time investment participants had to be willing to comply with, our sample may be particularly highly selective and homogenous. Overall, older age is characterized by great diversity and large individual differences in terms of health, as well as mental and social functioning. However, the variance of psychosocial functioning is likely reduced in our sample due to our strict inclusion criteria resulting in the selection of very homogenous and highly functioning participants. It may be that precisely this group of people does not show any effects of physical exercise on psychosocial functioning because they show very high levels of well-being initially, leaving little to no room for further improvement. Supporting this interpretation, studies have demonstrated that health benefits of physical activity are particularly successful in individuals who experience high levels of stress, as compared to low levels (Brown and Siegel, 1988; Carmack et al., 1999). While these findings suggest that physical activity could benefit health by ameliorating chronically high levels of stress, our participants showed low levels of perceived stress already at baseline. Additionally, several controlled studies that have identified the positive effects of exercise were conducted with clinical populations and not healthy, highly functioning older adults (e.g., Herring et al., 2010; Bridle et al., 2012).

Most studies investigating the effects of physical activity on psychosocial functioning have used questionnaires and have primarily tested between-person associations. Thus, in the current study and others with similar designs, there is an inherent need to employ methods that can examine dynamic within-person processes [for a review, see Kanning et al. (2013)]. The selected scales assessing well-being, perceived stress, loneliness, and future time perspective within the current intervention study may be not sensitive enough to measure within-person changes and may rather capture stable traits. The absence of exercise effects on well-being in the current healthy sample may result from a relatively high level of life satisfaction that older adults regularly experience, as well as the lack of sufficient sensitivity to small changes in the instruments used to measure subjective well-being.

Physical exercise can vary across various dimensions, further complicating the overall understanding of its relationship with psychosocial functioning. Different factors need to be assessed to gain a more global understanding of engagement in physical exercise and potential changes that can occur on the levels of physiology (e.g., catecholamines, endorphins), psychology (e.g., distraction, mastery), and environment (e.g., social integration). Our at-home training using bicycle ergometers and an app-based training regime may have presented different challenges to participants than training regimes in other studies. For example, physical exercise training was predominantly done at home in an individual setting, and there was only one group session per week. This provided participants with a great amount of flexibility as to when exactly to engage in the training, but at the same time put quite some responsibility on them to keep up with the training schedule. Similarly, there could be specific dose effects of exercise: overall duration of the intervention, but also individual session length and session frequency might have an influence on the psychosocial benefits. Meta-analyses targeting these questions in younger adults found that longer periods of training are associated with the largest effects on anxiety (Petruzzello et al., 1991) and depression (North et al., 1990). A review on training effects in the aging population concluded that programs lasting less than 10 weeks have less consistent effects on psychological well-being than longer study protocols (McAuley and Rudolph, 1995). However, at least regarding training duration, our intervention spanning 6 months was well beyond the 10-week threshold and should have provided enough time for an effect on psychosocial functioning.

However, it may also be that physical fitness *per se* is not implicated in physical effects on well-being. Several studies show effects of other training paradigms, such as dancing or yoga, on various psychological measures such as perceived stress and affect (West et al., 2004). Nevertheless, a common conception of the potential positive effects of physical exercise on brain structure and function, and therefore cognitive functioning, relies on the following assumed mechanism of action: physical exercise targets the cardiovascular system and may therefore lead to increased brain perfusion, resulting in better oxygenation of all cells (Pereira et al., 2007). This in turn may lead to maintained or even improved indices of brain structure, as angiogenesis may trigger neurogenesis, synaptogenesis, or changes in neuropil, all leading to increased gray matter volume (Kleemeyer et al., 2015; Maass et al., 2015). This line of reasoning is why much of the literature on plasticity in older adults, including our study, employs aerobic exercise to investigate its effect on psychological parameters. Whether this is indeed the most powerful tool to positively influence psychosocial factors in contrast to non-aerobic exercise training regimes has not been fully investigated. Less aerobically intensive forms of exercise may produce stronger effects on psychosocial functioning, as they cause less immediate stress for participants and may therefore exert a more direct effect on subjective well-being, for example. This is not to imply that the currently hypothesized mechanism of aerobic exercise on improved cognition and brain structure and function is invalid, but rather calls for a more differentiated investigation in the field regarding psychosocial functioning.

## Conclusion

Our 6-month at-home exercise intervention resulted in significantly increased fitness compared to a control group. Contrary to expectations, we did not find evidence for a beneficial effect of this fitness increase on psychosocial functioning, specifically on well-being, perceived stress, loneliness, or future time perspective. This may be due to pronounced stability of such psychological traits in older age; the current sample may have maintained relatively high levels of psychosocial functioning, which in turn may be due to the highly selective nature of this study. Finally, it is plausible that the mechanism underlying the beneficial effects of physical exercise on brain structure and function and on cognition differs fundamentally from that of the effects on psychosocial functioning. That is, while the positive effects on the brain and cognition are hypothesized to be driven by aerobic training *via* influences on brain perfusion, angiogenesis, and other cellular changes, the driving factors of change in psychosocial functioning may lie in other aspects of physical exercise, for example, in experiences of mastery or a feeling of community. This interpretation was suggested before (e.g., McAuley et al., 2000), and we can add that our findings are consistent with this interpretation, specifically, our finding that at-home bicycle ergometer interval training over 6 months did improve fitness but did not have a beneficial effect on the four major domains of psychosocial functioning we measured.

## Data Availability Statement

The raw data supporting the conclusions of this article will be made available by the authors, without undue reservation.

## Ethics Statement

The studies involving human participants were reviewed and approved by Ethikkommission der Deutschen Gesellschaft für Psychologie. The participants provided their written informed consent to participate in this study.

## Author Contributions

EW, SD, and SK: development study design. CM, EW, JP, SD, and SP: data acquisition. EW, JD, and SD: analyses. AB, BW, EW, SD, JD, JP, SK, and SP: evaluation of findings and writing the manuscript. All authors contributed to the article and approved the submitted version.

## Conflict of Interest

The authors declare that the research was conducted in the absence of any commercial or financial relationships that could be construed as a potential conflict of interest.

## Publisher’s Note

All claims expressed in this article are solely those of the authors and do not necessarily represent those of their affiliated organizations, or those of the publisher, the editors and the reviewers. Any product that may be evaluated in this article, or claim that may be made by its manufacturer, is not guaranteed or endorsed by the publisher.

## Footnotes

1
http://shared-reading.de/

2
https://jasp-stats.org

3
http://www.gpower.hhu.de/
