# Supplementary material for: No Evidence for a Boost in Psychosocial Functioning in Older Age After a 6-Months Physical Exercise Intervention
Source: Front Hum Neurosci. 2022 Mar 11;16:825454. doi: 10.3389/fnhum.2022.825454 (PMC8963719; doi:10.3389/fnhum.2022.825454)
Supplement: Supplementary file 1 [file Data_Sheet_1.PDF]

Supplementary Material for „No boost in psychosocial functioning in older age after a six-months physical exercise intervention“

Bayesian repeated measures ANOVAs
With factors time (pre vs. post) and group (no-sport vs. sport)

VO2peak

Model Comparison

Models	P(M)	P(M|data)	BF_M	BF_{10}	error %
time + group + time ∗ group	0.200	0.937	59.200	1.000	
time + groupt	0.200	0.033	0.135	0.035	11.424
time	0.200	0.031	0.127	0.033	11.175
group	0.200	1.357e-5	5.429e-5	1.449e-5	11.519
Null model (incl. subject)	0.200	1.255e-5	5.022e-5	1.340e-5	11.141

Note. All models include subject

Analysis of Effects	P(incl)	P(excl)	P(incl|data)	P(excl|data)	BF_{incl}
time	0.600	0.400	1.000	2.613e-5	25514.883
group	0.600	0.400	0.969	0.031	21.043
time ∗ group	0.200	0.800	0.937	0.063	59.200

Well-being

Model Comparison

Models	P(M)	P(M|data)	BF_M	BF_{10}	error %
Null model (incl. subject)	0.200	0.628	6.739	1.000	
group	0.200	0.197	0.982	0.314	2.880
time	0.200	0.124	0.566	0.197	1.989
time + group	0.200	0.039	0.162	0.062	3.696
time + group + time ∗ group	0.200	0.013	0.052	0.020	4.286

Note. All models include subject

Analysis of Effects	P(incl)	P(excl)	P(incl|data)	P(excl|data)	BF_{incl}
time	0.600	0.400	0.175	0.825	0.142
group	0.600	0.400	0.249	0.751	0.221
time ∗ group	0.200	0.800	0.013	0.987	0.052

Perceived stress

Model Comparison

Models	P(M)	P(M|data)	BF_M	BF_{10}	error %
Null model (incl. subject)	0.200	0.561	5.114	1.000	
group	0.200	0.224	1.157	0.400	0.736
time	0.200	0.142	0.664	0.254	2.584
time + group	0.200	0.056	0.237	0.100	1.821
time + group + time ✻ group	0.200	0.016	0.066	0.029	1.843

Note. All models include subject

Analysis of Effects	P(incl)	P(excl)	P(incl|data)	P(excl|data)	BF_{incl}
time	0.600	0.400	0.214	0.786	0.182
group	0.600	0.400	0.296	0.704	0.281
time ✻ groupt	0.200	0.800	0.016	0.984	0.066

Loneliness

Model Comparison

Models	P(M)	P(M|data)	BF_M	BF_{10}	error %
Null model (incl. subject)	0.200	0.413	2.809	1.000	
time	0.200	0.347	2.125	0.841	1.012
group	0.200	0.118	0.534	0.286	2.742
time + group	0.200	0.101	0.451	0.246	3.397
time + group + time ✻ group	0.200	0.021	0.088	0.052	4.103

Note. All models include subject

Analysis of Effects	P(incl)	P(excl)	P(incl|data)	P(excl|data)	BF_{incl}
time	0.600	0.400	0.470	0.530	0.590
group	0.600	0.400	0.241	0.759	0.211
time ✻ group	0.200	0.800	0.021	0.979	0.088

Future time perspective

Model Comparison

Models	P(M)	P(M|data)	BF_M	BF_{10}	error %
time	0.200	0.603	6.080	1.000	
time + group	0.200	0.203	1.017	0.336	3.647
Null model (incl. subject)	0.200	0.103	0.460	0.171	0.856
time + group + time ✻ group	0.200	0.054	0.229	0.090	4.377
group	0.200	0.037	0.152	0.061	2.982

Note. All models include subject

Analysis of Effects	P(incl)	P(excl)	P(incl|data)	P(excl|data)	BF_{incl}
time	0.600	0.400	0.860	0.140	4.100
group	0.600	0.400	0.294	0.706	0.277
time ✻ group	0.200	0.800	0.054	0.946	0.229
